# Seroprevalence and risk factor analysis of brucellosis among dairy farmers in Aligarh region, North India: creating awareness of a neglected disease

**DOI:** 10.1099/acmi.0.000648.v3

**Published:** 2024-03-25

**Authors:** Alveera Alam, Hiba Sami, S. Zeeshan Ahmad Hashmi, K. Gururaj, Mohammad Azam Khan, Parvez A. Khan, Haleema Ahmad, Nazish Fatima, Haris M. Khan

**Affiliations:** 1 Jawaharlal Nehru Medical College, Aligarh Muslim University, Aligarh, India; 2 Department of Microbiology, Jawahar Lal Nehru Medical College, AMU, Aligarh, India; 3 Animal Health Division, ICAR-CIRG, Makhdoom, Farah, Mathura – 281122, Uttar Pradesh, India; 4 Department of Statistics and OR, AMU, Aligarh, India

**Keywords:** awareness, brucellosis, dairy farmers, neglected disease

## Abstract

**Introduction.** Brucellosis, a globally distributed zoonotic disease, is caused by the Gram-negative bacteria known as *Brucella*. Humans acquire infection through direct contact with the blood, urine and placenta of animals, inhalation of dust or aerosols at infected animal farms, and raw milk and meat intake. This study aimed to assess the prevalence of brucellosis in dairy farmers in and around the Aligarh region of North India, to document various clinical signs and symptoms in *Brucella-*positive individuals, and to create awareness in dairy farmers concerning brucellosis and ways to prevent it.

**Methods.** This was an observational study that included 125 dairy farmers in and around the Aligarh region. Serum samples were taken from this high-risk group after obtaining informed consent. Further, a pre-designed proforma was used to collect information about their knowledge, attitude and practices (KAP) concerning brucellosis and assess the risk factors for the disease. The Rose Bengal test (RBT), serum agglutination test (SAT) and enzyme-linked immunosorbent assay (ELISA) were performed to detect the seroprevalence of brucellosis.

**Result.***Brucella* infection was diagnosed in 64 (51.20 %) cases by indirect ELISA (IgM+IgG), 41 (32.8 %) by RBT and 4 (3.2 %) by SAT. Significant clustering of patients was seen in the 20–55 years age group. The most common symptoms in ELISA IgM-positive patients were joint pain (16.07 %), fatigue (14.28 %), anorexia (12.50 %), weight loss (8.92 %), malaise (5.35 %), undulant fever (3.57 %), night sweats (3.57 %) and headache (1.78 %). The findings of this study indicate that ELISA (IgM+IgG) exhibits great sensitivity as compared to SAT and RBT. KAP was very poor among dairy farmers.

**Conclusion.** In India, *Brucella* is a frequent but severely underreported illness. ELISA is the most sensitive serological test for diagnosing brucellosis. No potential vaccine has yet been introduced for humans against brucellosis. Thus, it is necessary to impart awareness and sensitize high-risk groups concerning brucellosis.

## Data Summary

The data are available in the paper. No external source of data has been used.

## Introduction

The Gram-negative bacteria *Brucella* are the source of the zoonotic illness brucellosis, which is common and widespread [1]. Malta fever, Mediterranean fever, Gibraltar fever, Cyprus fever, undulant fever, or typhomalarial fever are some of the other names for it [2]. Brucellosis in humans begins as a crippling acute illness that can develop into a chronic condition with several problems. *Brucella* species include *Brucella abortus*, *Brucella melitensis*, *Brucella suis*, *Brucella canis*, *Brucella ovis*, *Brucella neotaomae*, *Brucella microti*, *Brucella ceti* and *Brucella pinnipedalis* [3].

Human brucellosis is more common in regions where animal brucellosis is endemic. It is endemic in countries of the Mediterranean basin, Central Asia and China, the Indian Subcontinent, the Middle East, and Central and South America [2]. Each year, more than 500 000 human cases of brucellosis are recorded worldwide. The prevalence of brucellosis in different areas of the world has risen due to a focus on higher animal production and aggregation under unsanitary circumstances and this is especially true for the dairy production sectors that have sprouted up near fast-rising metropolitan centres in many emerging nations, including India.

Animal, managerial, occupational and environmental variables; consuming raw meat, but mostly unpasteurized milk and cheese from diseased animals; and contact with placentas from aborted foetuses, are risk factors for human brucellosis infection. Age, breed and sex of the animal; a history of retained placenta and miscarriage; parity; and milking methods are animal variables. Environmental variables are mostly connected with the animals' agroecological position in either endemic or brucellosis-free areas [4]

In India, ~70 million households are involved in dairy farming of which ~15 % own more than 4 milch animals [5]. Many studies report a high prevalence of brucellosis among dairy farmers. Consumption of raw milk and uncooked meat from cattle unvaccinated for brucellosis, purchasing brucellosis-infected cattle, and cotact during labour and handling aborted foetuses without using protective gear such as gloves and aprons are some of the risk factors for brucellosis observed in dairy farmers [6]. Because of its vast array of vague clinical manifestations that can persist from around a few days to more than a year, brucellosis is sometimes mistaken for more widespread illnesses, such as typhoid fever, malarial fever, etc. The most typical symptoms are frequent episodes of fever, exhaustion, chills, arthralgia, increased night time sweating, malaise and headache. The fever is of the ‘undulating form’ – this means that there will be afebrile periods in between feverish intervals, which might extend for weeks on end. Vertebral osteomyelitis commonly involves the lumbar and lower thoracic vertebrae, while joints such as the knee, hip, sacroiliac and shoulder joints are commonly affected in septic arthritis. Patients may also develop hepatomegaly, splenomegaly, or lymphadenopathy. The objective of this study was to assess the prevalence of brucellosis in dairy farmers in and around the Aligarh region of North India, to document various clinical signs and symptoms in *Brucella-*positive individuals, and to create awareness in dairy farmers concerning brucellosis and discuss ways to prevent it.

## Methods

### Study design

This was a descriptive, cross-sectional study.

### Study area

This study was carried out in Aligarh and adjoining areas of Uttar Pradesh.

### Study period

The study was performed over a 3 months period from August to October 2022.

### Inclusion criteria

The patients were willing to give informed consent.

### Exclusion criteria

1. Patients refusing to give informed consent.
2. Patients with a history of tuberculosis.
3. Patients with obvious clinical signs for diseases such as diarrhoea, pneumonia, urinary tract infection (UTI), typhoid fever and malaria are excluded from the study.
4. Patients receiving antiretroviral treatment (ART) or antitubercular treatment (ATT).

### Study population

The study population consisted of 125 dairy farmers. Individuals spending maximum time with cattle were approached. Those who gave consent to participate were surveyed by documenting their responses in a pre-designed proforma and blood samples were collected to run serological tests to determine the prevalence of the disease brucellosis. As samples were collected from the participants only once, the attrition rate was not calculated. Samples comprising 5–10 ml of venous blood were drawn to run serological tests.

Close-ended questionnaires with two sections were utilized to obtain data. The first part of the survey asked about sociodemographic data, including age, sex and degree of education. A modified Kuppuswamy scale [7] was used for classification of population according to different socioeconomic status. The route of disease transmission from animal to human, routine procedures for handling animals and animal products, and the respondent’s dietary habits were all included in the second section of the questionnaire regarding the respondent’s risk factors for brucellosis. The following serological tests were performed for serological diagnosis.

#### Rose Bengal test (RBT)

**Fig. 1.**
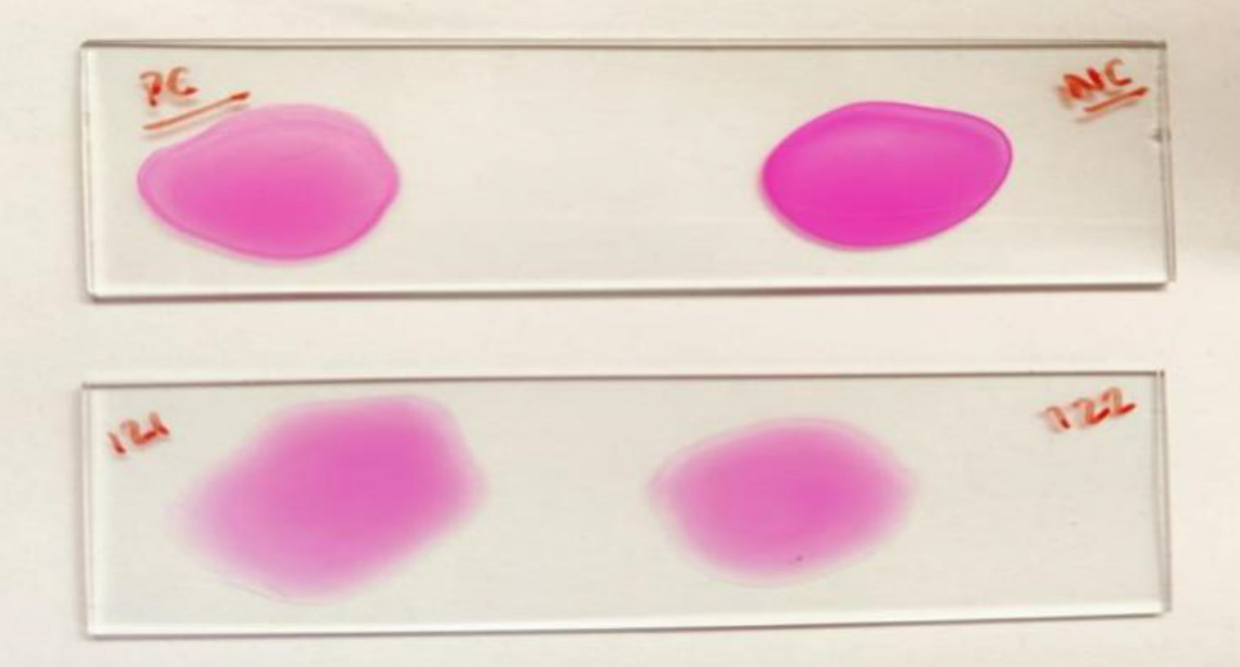
RBT for human brucellosis.

RBT is a rapid plate agglutination test widely used in the diagnosis of human brucellosis. It confers a high degree of sensitivity as it detects agglutinating and non-agglutinating antibodies and avoids the prozone phenomenon. Samples were serially diluted twice in 0.9 % normal saline solution. Each circle received one drop of BRUCEL-RB antigen suspension, and the contents of each circle were mixed equally across the whole circle using different mixing sticks. Formation of agglutinate within 4 min with antigen was recorded as a positive reaction. RBT was performed using BRUCEL-RB antigen (Fig. 1).

#### Serum agglutination test (SAT)

All samples were tested by standard tube agglutination test SAT [8] according to the standard procedure. The highest serum dilution at which 50 % agglutination was observed was marked as the endpoint for titre. A titre of 1 : 160 and above was regarded as serologically positive. SAT was performed on all samples using standard procedures.

#### Enzyme-linked immunosorbent assay (ELISA)

**Fig. 2.**
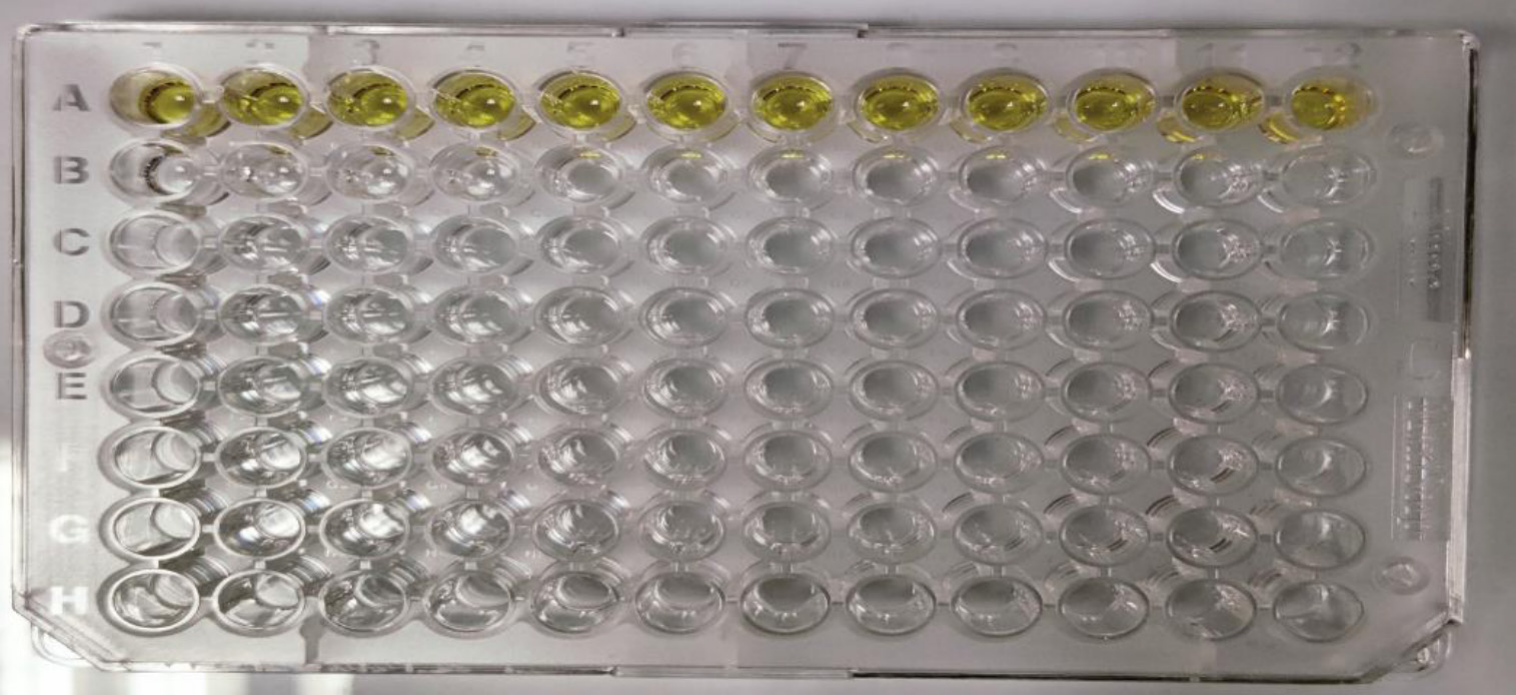
ELISA plate after standardization run for antigen.

ELISA was performed for IgM and IgG antibodies on all the samples using an ELISA kit and results were interpreted accordingly. ELISA for brucellosis was performed at Central Institute for Research on Goats (CIRG), Makhdoom, PO Farah, Mathura, Uttar Pradesh, India. Indirect ELISA (iELISA) as performed by Rizvi *et al*. [9] was used for the study. For the above procedure in-house synthesized antigen of *Brucella* at a concentration of 1 mg ml^−1^ was used. Standardization of the antigen was attained using standard procedures (Fig. 2). The cut-offs detailed in Table 1 were used for the diagnostic criteria in our study.

**Table 1.** Cut-offs used in iELISA

SP ratio	iELISA interpretation	Result/status of infection
0.00–0.09	Negative (N)	
0.10–0.24	Suspected or Borderline (S)	Negative
0.25–0.39	Low positive (LP)	
0.40–0.99	Positive (P)	Positive
1.00–10.0	Strongly positive (SP)	

Formula S/P ratio = (OD of sample − OD of negative control) / (OD of positive control − OD of negative control) (OD stands for the optical density which is the absorbance at 450 nm).

### Data collection and statistical analysis

The data collected in the study were coded and transferred to a personal computer and analysed using SPSS Windows version 11. Statistical tests such as the chi-square test and Fisher’s exact test were used. The demographic and clinical variables collected were used to stratify study groups and were incorporated into multivariate analysis models to identify confounders or true associations. Statistical significance was accepted at the confidence level of 95 %.

### Ethical consideration

Ethical clearance from the institutional ethical committee was obtained prior to the study. The nature of the study was fully explained to the participants and written informed consent was obtained from them.

### KAP

In order to create awareness, a questionnaire was used to gather information on dairy farmers’ knowledge, attitudes and practices concerning *Brucella*.

## Results

The study included 125 dairy farmers, the majority of whom belonged to the age group 30–50 years. According to the data, out of 125 samples, 64 (51.20 %) were ELISA-positive, 41 (32.8 %) were RBT-positive and 4 (3.25 %) were SAT-positive (Table 2).

**Table 2.** Comparison of results for indirect ELISA, SAT and RBT in detecting human brucellosis

	ELISA IgM+IgG	SAT	RBT
*Brucella*-positive cases	64 (51.2 %)	4 (3.25 %)	41 (32.8 %)

**Fig. 3.**
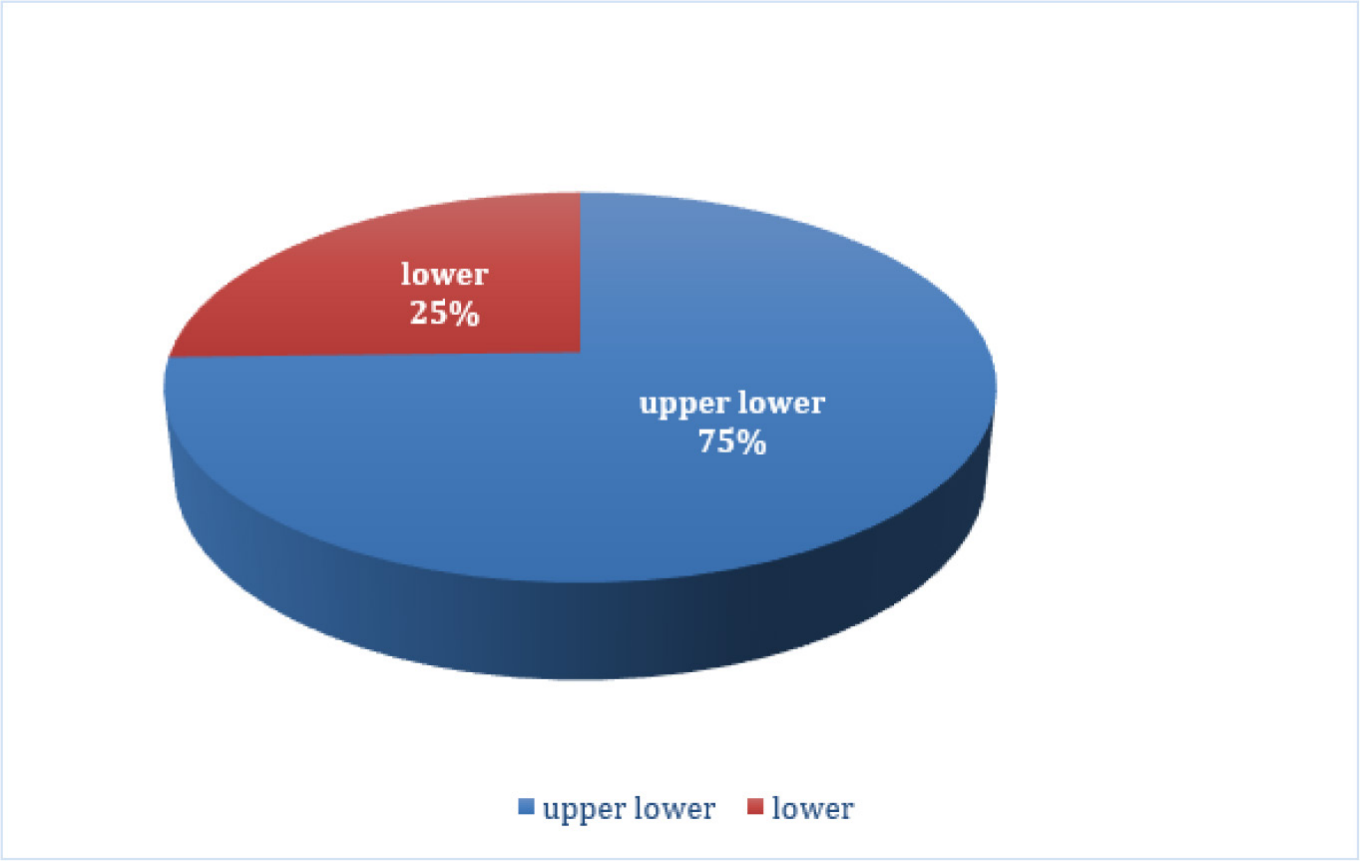
Socioeconomic distribution of *Brucella*-positive dairy farmers according to the modified Kuppuswamy scale.

Seventy per cent of the *Brucella*-positive dairy farmers belonged to the upper lower class, while 25 % belonged to the lower class (Fig. 3). A modified Kuppuswamy scale was used to calculate the data.

Table 3 shows the area-wise distribution of ELISA-positive dairy farmers. The majority of positive individual belonged to Silla followed by Taleypur and Madhavgadh.

**Table 3.** Area-wise distribution of *Brucella*-positive individuals (*n*=64)

		Positive	Negative	*P* value
	Mirzapur	6	6	
	Green Park Colony	1	1	
	Taleypur	10	14	
	Maheshpur	0	4	
Area of residence	Shahjamal	2	2	chi=11.972 *P*=0.366
	Saperabahanpur, Jawan Block	6	3	
	Badarbagh	4	2	
	Manjoorgadhi	5	1	
	Silla	11	8	
	Rathgawan	5	4	
	Madhavgadh	10	7	
	Saungra	4	9	

The age range was 10–90 years. Those with brucellosis were distributed as follows: 0–30 years (*n*=26), 31–40 years (*n*=27) and >40 years (*n*=72). The highest prevalence was seen in the age group 0–30 years (80.07 %), followed by 31–40 years (48.14 %) and >40 years (41.66 %). The prevalence of brucellosis was found to be higher in females (63.63 %) than in males (46.73 %). (Table 4).

**Table 4.** Demographic characteristics of seropositivity among dairy farmers (*n*=64)

Characteristics	Category	Positive	Tested	Prevalence	*P* value
**Age**	0–30	21	26	80.07%	chi=11.818*P*=0.003
	31–40	13	27	48.14%	
	>40	30	72	41.66%	
**Gender**	Female	21	33	63.63%	chi=19.748*P*=0.0001
	Male	43	92	46.73%	
**Socioeconomic status**	Lower	16	37	43.24%	chi=1.332*P*=0.249

The correlation between brucellosis and several risk factors is shown in Table 5. A statistically significant correlation existed between interactions with animals suffering from reduced milk yield and human brucellosis, with a prevalence of 22.76 % (OR 1.31, RR 1.15), followed by mastitis, with a prevalence of 20.00 % (OR 1.35 RR 1.14). The prevalence of recurrent abortion was 15.44 % (OR 1.48, RR 1.15), high temperature was 1.63 % (OR 1.32 RR 1.29), birth of weak/non-viable calves was 10.40 % (OR 0.23 RR 1.17) and hygroma was 6.40 % (OR 0.99 RR 0.955). Sterility was not found in any animal.

**Table 5.** Multivariate analysis of risk factors for brucellosis (*n*=64)

Risk factors in animals	ELISA-positive individuals	Total no. of dairy farmers tested	Prevalence (%)	Odds ratio (95 % CI)	Relative risk
Recurrent abortion	19	125	15.44 %	1.36	1.15
Reduced milk yield	28	125	22.76 %	1.31	1.13
High temperature	2	125	1.63 %	1.87	1.29
Birth of weak/non-viable calves	13	125	10.40 %	1.42	1.17
Sterility	0	125	0 %	0.467	0
Hygroma	8	125	6.40 %	0.911	0.955
Mastitis	25	125	20.00 %	1.35	1.14

**Fig. 4.**
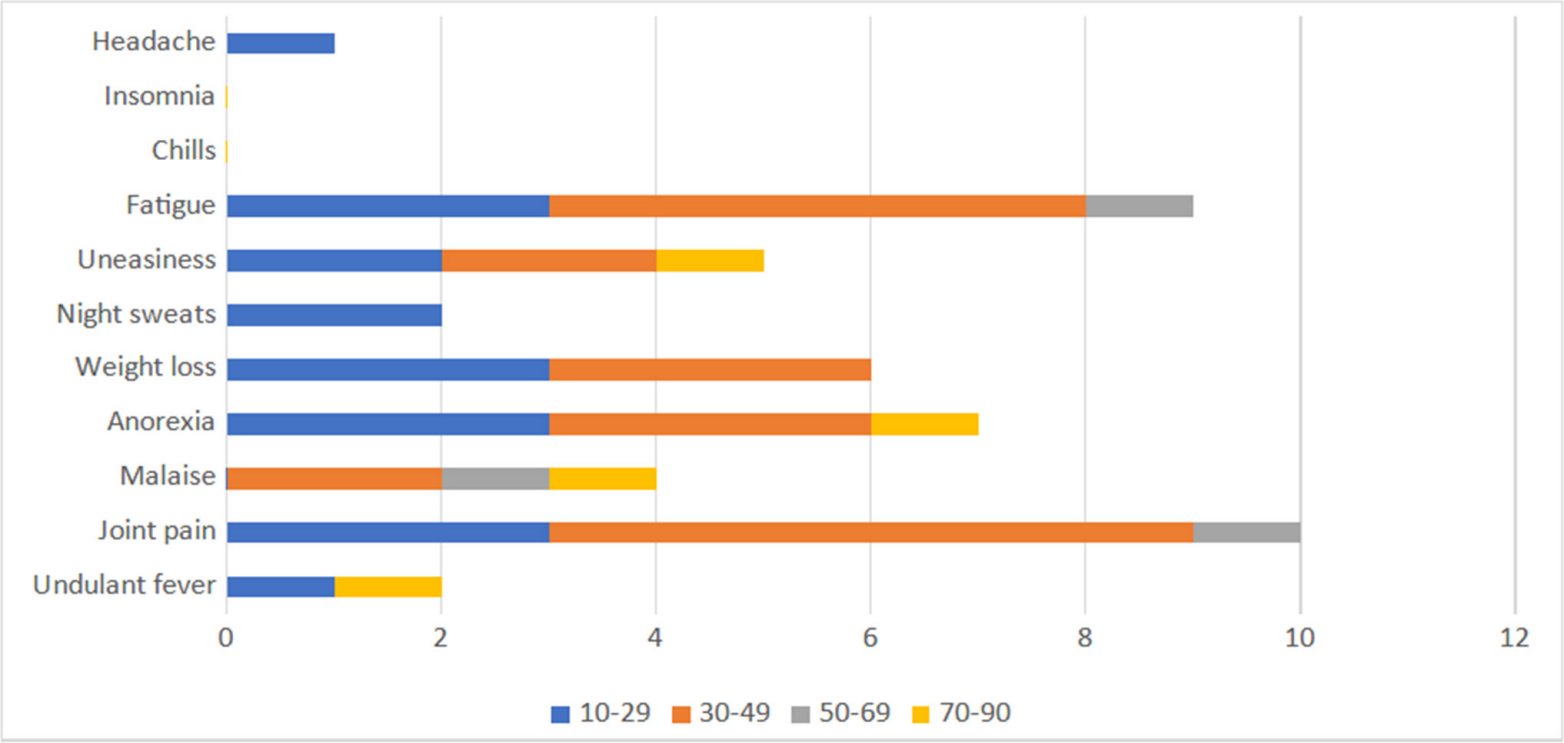
Distribution of clinical manifestations in *Brucella*-positive individuals in various age groups (*n*=64).

When clinical manifestations from different age groups were compared, the age range of 30–49 years showed the highest prevalence of joint pain and fatigue. Undulant fever was prevalent in the age range of 10–29 years and 70–90 years (Fig. 4).

**Fig. 5.**
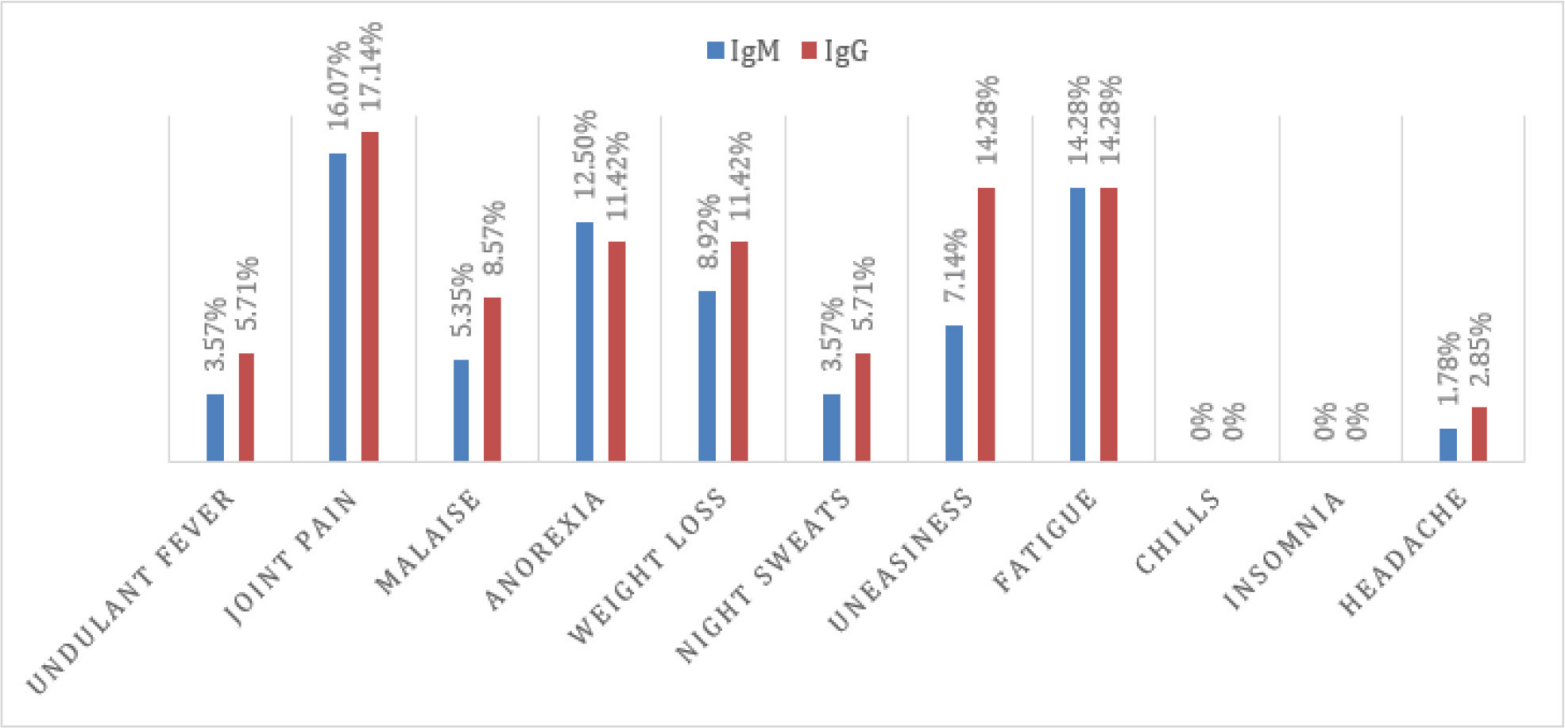
Comparison of symptoms of brucellosis between IgM- and IgG-positive individuals.

The most common symptom discovered was joint pain, with an ELISA IgM prevalence of 16.07 % and an ELISA IgG prevalence of 17.14 %. This was followed by undulant fever, which had an ELISA IgM prevalence of 3.57 % and an ELISA IgG prevalence of 5.71 %. Other symptoms that were documented include anorexia, malaise, weight loss, night sweats, uneasiness and fatigue. Both ELISA IgM and IgG found that symptoms were more or less equally prevalent (Fig. 5).

**Fig. 6.**
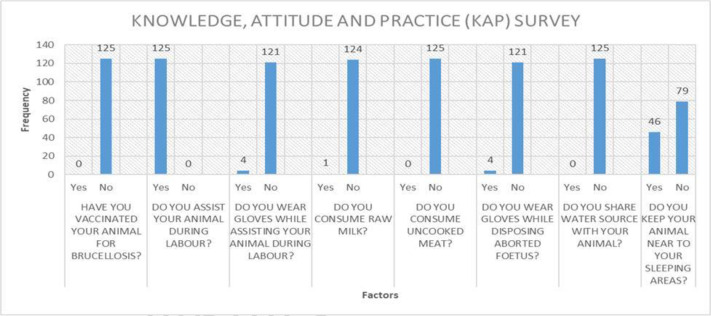
KAP Survey and assessment of risk factors associated with human brucellosis (*n*=125).

None of the patients who participated in the study has knowledge of brucellosis. None of the participants in the study vaccinated their animals against brucellosis. Only 3 of the 125 participants wore gloves while supporting their animals during childbirth and 46 of 125 participants keep their animal near to their sleeping areas. Fig. 6 shows the KAP of dairy farmers concerning brucellosis awareness.

## Discussion

Brucellosis has been recognized as a re-emerging disease in underprivileged countries throughout the world, including India, as a serious zoonotic infection. According to this study, dairy producers had a greater incidence of brucellosis, with a prevalence of 51.20 %. In fact, the stated figure was likely significantly lower than the real number because under-diagnosis and under-reporting of brucellosis are acknowledged issues in many underdeveloped nations. It is estimated that for every recorded case, there are at least two more instances that are not reported or are not diagnosed [10].

Among 125 subjects, 40 subjects were not positive for either of the tests – ELISA IgG/IgM, RBT or SAT – and 9 samples were positive for all the four tests – ELISA IgG, ELISA IgM and RBT. Four (3.2 %) SAT cases, 41 (32.8 %) RBT cases and 64 (51.20 %) instances of indirect ELISA (IgM+IgG) were positive for *Brucella*. Similar findings were found in different research [11], which demonstrated that indirect ELISA was the most sensitive serological test for finding brucellosis.

A high prevalence of brucellosis was observed in females (63.63 %) as compared to males (46.73 %). The most likely reason for this is occupational exposure to animals. According to a study by Alusi *et al*. [12], men are in charge of aiding animals during reproduction, whilst females play a significant part in carrying out everyday tasks such as milking animals. Similar results were seen in the current study, which revealed that the females are engaged in routine livestock care and men are more in charge of aiding in cow reproduction [12]. Additionally, we discovered in our study that coming into contact with an animal whose milk yield had decreased carried the highest risk. This accounts for the greater prevalence in females.

Human brucellosis was seen in this research in all age categories (10–90 years), but it was mostly concentrated in those aged 20 to 55 years. The largest prevalence of 80.07 % was seen in the age group 10–30 years. This is supported by another study that shows that the majority of dairy farmworkers are adults, with the majority of them falling within the age range of 18 to 35. This is most likely because farming requires hard manual labour [13].

Brucellosis exposure was greater among dairy producers who work with animals that produce less milk (22.76 %). Some infected cows may not become serologically positive until later in the pregnancy and after giving birth [14]. This suggests a risk of infection for livestock owners who often interact with and have a tradition of drinking unpasteurized milk and milk products. Direct contact with animals suffering from recurrent abortion (15.44 %), mastitis (20.00 %), hygromas (6.40 %), high temperature (1.63 %) or giving birth to weak and non-viable calves (10.40 %) has also been recognized as a key risk factor. Brucellosis has been linked to the pregnancy stage that comes before milking. This is because *Brucella* species have a preference for the uterus of the pregnant animal because the placenta produces the sugar erythritol, which the organism preferentially metabolizes [14]. It seems to make sense that helping animals give birth raises the risk of infection, given that *Brucella* spp. are known to prefer reproductive tissues, notably the placenta, and aborted foetuses. This is corroborated by the evidence that touching parturient products and aiding animals during abortions both enhance the chance of contracting brucellosis and aid in its spread [15].

Brucellosis has a wide spectrum of clinical indicators and can last for days or even years; nonetheless, it is commonly misinterpreted, resulting in inadequate therapy and prolonged sickness. The most common signs and symptoms found in this research were joint pain and fatigue, followed by anorexia, weight loss and fever. Other studies [16,17] have also reported fatigue and arthralgia as the most common symptom of brucellosis in humans, followed by fever, back pain and loss of appetite. When clinical symptoms from various age groups were evaluated, it was shown that joint pain and fatigue were more common in those between the ages of 30–49. In the 10–29 and 70–90 age groups, undulant fever was common. Weight loss was significantly seen in the 10–49 years of age group and night sweats were only observed in the 10–29 age group. Most of the symptoms were found in the 10–29 years of age group.

Around 75 % of the population considered in the study belonged to the upper lower class, while the remaining 25 % belonged to the lower class. This shows that most dairy producers were raising their cows according to traditional knowledge and lacked proper training. The illness’s societal impact includes major human healthcare expenditures for diagnosis and treatment, as well as non-healthcare costs such as public education initiatives to decrease disease transmission

Regarding the KAP (Fig. 6) among dairy producers, all 125 respondents showed no understanding of the illness, and none of the participants had given their animals brucellosis vaccinations. This indicates an urgent need to familiarize dairy farmers in India with brucellosis. Another study among dairy farmers from India [18] also showed that only 3.4 % of dairy farmers from Assam and Bihar had some idea of brucellosis and 91.9 % of them had never heard of it.

This study educated dairy farmers about preventative measures and raised their knowledge of them. They were instructed to handle livestock in a sanitary manner. Participants received sensitization concerning the warning signs and symptoms of *Brucella*-infected cattle as well as human symptoms, the disease’s route of transmission, and the animal vaccine. They were told to drink pasteurized milk and to stay away from raw meat. They were told to put on gloves, gowns and aprons when handling cows during labour, disposing of diseased animals' aborted foetuses, and so on. They were urged to take part in any brucellosis monitoring work, whether it included humans or animals.

## Conclusion

This research backs up the idea that dairy farmers are at higher risk of developing brucellosis. Despite a considerable population being exposed to risk factors with a concomitant prevalence of brucellosis, it is still a neglected illness in India. This may be a result of the disease’s vague symptoms. As direct or indirect contact with infected animals or their products is the main cause of human brucellosis, prevention must be centred on cutting off this unsanitary interaction. Sensitization of the farming community is a crucial initial step in preventing the disease, since many underdeveloped and developing countries such as India lack the personnel and financial resources required to totally eradicate the sickness from animals.

## Limitations

As the KAP was assessed using a questionnaire and no direct observations were made, there is a possibility of recall bias.

## Abbreviations

ELISA: enzyme-linked immunosorbent assay
OD: optical density
RBT: rose bengal test
SAT: serum agglutination test
